# Pre-Whaling Genetic Diversity and Population Ecology in Eastern Pacific Gray Whales: Insights from Ancient DNA and Stable Isotopes

**DOI:** 10.1371/journal.pone.0035039

**Published:** 2012-05-09

**Authors:** S. Elizabeth Alter, Seth D. Newsome, Stephen R. Palumbi

**Affiliations:** 1 Department of Biological Sciences, Hopkins Marine Station, Stanford University, Pacific Grove, California, United States of America; 2 Department of Zoology and Physiology, University of Wyoming, Laramie, Wyoming, United States of America; University of Otago, New Zealand

## Abstract

Commercial whaling decimated many whale populations, including the eastern Pacific gray whale, but little is known about how population dynamics or ecology differed prior to these removals. Of particular interest is the possibility of a large population decline prior to whaling, as such a decline could explain the ∼5-fold difference between genetic estimates of prior abundance and estimates based on historical records. We analyzed genetic (mitochondrial control region) and isotopic information from modern and prehistoric gray whales using serial coalescent simulations and Bayesian skyline analyses to test for a pre-whaling decline and to examine prehistoric genetic diversity, population dynamics and ecology. Simulations demonstrate that significant genetic differences observed between ancient and modern samples could be caused by a large, recent population bottleneck, roughly concurrent with commercial whaling. Stable isotopes show minimal differences between modern and ancient gray whale foraging ecology. Using rejection-based Approximate Bayesian Computation, we estimate the size of the population bottleneck at its minimum abundance and the pre-bottleneck abundance. Our results agree with previous genetic studies suggesting the historical size of the eastern gray whale population was roughly three to five times its current size.

## Introduction

Commercial whaling in the 19^th^ and 20^th^ centuries resulted in greatly reduced population sizes in many species, with dramatic impacts on marine ecosystems (e.g. [1]). Despite widespread scientific and public interest in the recovery of whale stocks and the ecological impacts of removal, little is known about how whaling may have altered basic aspects of population ecology including abundance, foraging grounds, migration patterns, or population substructure [2], [3].

Of particular interest is the estimation of historic abundance immediately prior to whaling. Genetic diversity in many whale populations is too high to match pre-whaling population sizes estimated from whaling and commercial records, producing a striking discrepancy between historic abundance in baleen whales estimated from historical records versus genetic data (e.g. [4], [5]). For example, mitochondrial data from three baleen whale species in the North Atlantic produced estimates 6 to 20 times larger than previous estimates based on historical data [4]. Many potential explanations for this discrepancy have been suggested [6]. For example, abundances estimated from historical data could be too low if whaling records were lost, biased or falsified, or if parameters (such as struck-and-lost rate) used to calculate the numbers of whales killed from these records are inaccurate. On the other hand, abundances from genetic data could be too high if the mutation rate used is too low, if few genetic markers were used, if population structure is not accounted for, if generation time is underestimated, or if balancing selection was occurring at the genetic loci used to calculate population size. Many of these factors have been and continue to be investigated as sources of error (see [6], [7]).

However, the discrepancy between historic and genetic estimates can also be explained by a single scenario: populations of whales were much larger in the past, but declined substantially before whaling began. Under this scenario, both genetic and historic inferences could be correct. However, this hypothesis has proven difficult to test, as it requires estimation of prehistoric population dynamics.

Ancient DNA sequences allow direct estimation of changes in genetic diversity over time, and can greatly improve the reconstruction of historic population dynamics, particularly when demographic histories are complex [8], [9]. Temporally-spaced genetic data can improve statistical power to detect bottlenecks relative to modern data alone, even when relatively few ancient samples are available [10]. Demographic reconstruction using ancient sequences has yielded insight into historic population ecology and the context of declines in organisms such as bison [11], woolly mammoths [12], and tuco tuco [13], and has the potential to provide information about the historical demography of whales before whaling. Ancient genetic data can be particularly powerful when combined with stable isotope data, which can reveal information about feeding ecology from the same population [14], [15].

In this study, we investigate the pre-whaling genetic diversity, population dynamics and feeding ecology of the eastern Pacific gray whale using ancient and modern DNA sequences and stable isotope data. Eastern gray whales represent a useful case study for investigating historic population dynamics and in particular the discrepancy between genetic and historical data, because both genetic diversity and historical records have been examined in depth [5], [16], [17]. According to historic records, eastern Pacific gray whales originally numbered around 15,000–20,000 individuals before whaling [16]; modeling based on census data extends these numbers to 19,500–35,500 individuals [18]. Intensive whaling from 1850 to 1874 and subsequently from the turn of the century until the 1930s reduced this population to some unknown fraction of its former size. In contrast, estimates from multilocus genetic data are consistent with a much higher original population size (78,000–116,000 individuals) [5].

A pre-whaling bottleneck in gray whales could have several potential causes. Because they feed in Arctic and subarctic benthic environments, gray whales are thought to be relatively sensitive to changes in climate, and climatic events such as the Medieval Warm Period (ca. 900–1200 AD) or Little Ice Age (ca. 1300–1850 AD) could have caused a population decline. The nature of the relationship between gray whale populations and climate-sensitive ecosystem features such as sea ice, freshwater input to nearshore benthic ecosystems and benthic species composition is poorly understood [19], [20]. However, recent calving rates have been shown to be negatively correlated with ice cover extent, indicating population growth is faster when ice cover is reduced and feeding habitat is extended [20]. Indigenous hunting of gray whales has been occurring for at least 5000 years around the Pacific Rim and could have reduced gray whale populations below original levels. Though it has always been assumed that hunting using traditional techniques had minimal impact on whale abundance [21], the actual effects of indigenous hunting have not been quantified. A final possibility is that killer whales (*Orcinus orca*), the major predator on gray whales, may have increased or switched to feeding primarily on gray whales (e.g. [1]).

The accurate inference of population dynamics from ancient sequences requires multiple, well-dated samples from a single population, and depends on a number of assumptions related to the coalescent including random selection of individuals from a panmictic population [22]. We utilized whale bones excavated from dated archaeological sites on the Makah and Quilleute tribal reservations, dated 150–3500 years before present (ybp). To detect a pre-whaling bottleneck, we used genetic data from these dated ancient samples along with a modern gray whale dataset in two different and complementary analyses: 1) serial coalescent simulations with approximate Bayesian computation to determine posterior probability distributions for demographic parameters; and 2) a Bayesian MCMC method [8], which uses a coalescent approach to compare the likelihood of different histories.

In addition to investigating genetic diversity of modern and ancient samples, we used stable isotope analysis to investigate how feeding ecology may have changed since whaling, particularly around the Olympic peninsula and Vancouver Island. Today, most gray whales feed in the Bering, Chukchi and Beaufort Seas, though a small number of “summer residents” are known to feed near Vancouver Island and other locations in the Pacific Northwest (e.g. [23]). Abundant bones found in archaeological sites around the Chukchi peninsula (e.g. [24]) suggest the majority of gray whales fed in the Bering Sea and northward in the past. However, the larger population size of gray whales before whaling may also have resulted in alternative foraging habitats or strategies. In particular, productive areas in the Pacific Northwest including the inlets and sounds of Vancouver Island may have supported sizeable feeding populations [25]. Stable isotope analysis, particularly carbon (δ^13^C) and nitrogen (δ^15^N), can be used to distinguish between marine foraging areas on a broad geographic scale (reviewed in [15], [26]), and thus can be used to determine whether the ancient gray whales from the Pacific Northwest represented a local feeding group. Because the samples used in this study come from the same region as the modern feeding agreggation of gray whales in the Pacific Northwest, we compared stable isotope (δ^13^C and δ^15^N) values between ancient and modern samples to determine whether ancient samples were derived from individuals representing a local feeding subpopulation.

## Materials and Methods

### Samples

**Figure 1:**
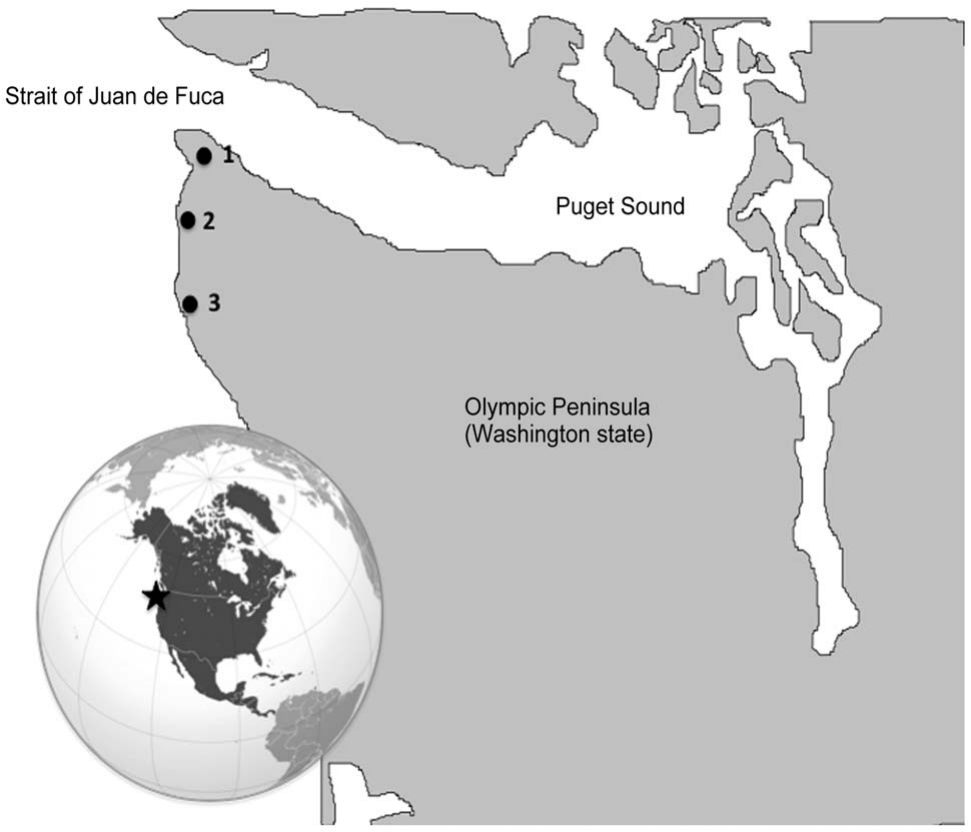
Sampling locations for archaeological material on the Olympic Peninsula, Washington state, USA. 1 = Shell midden deposit on Makah Tribal Reservation (45CA400); 2 = Ozette site (45CA24B70); 3 = Shell midden deposit on Quilleute Tribal Reservation (45CA23). Samples were excavated between 1971 and 2005 [28].

Modern mitochondrial control region sequences from 120 eastern Pacific and 45 western Pacific gray whales were obtained from NCBI [17]. These datasets are comprised of samples from both stranded individuals across the migratory route (eastern Pacific) and biopsies (western Pacific) across numerous years. Subsequent sampling in the eastern Pacific population [3] found essentially the same distribution of mitochondrial haplotypes as in [17], suggesting this dataset contains a reasonable representation of the haplotype distribution in the population. Forty-two of these samples were reamplified and sequenced in our laboratory and sequenced blind in both directions (see [27] for methods), and sequences were compared with those from NCBI. Subsamples of 40 whale bones were collected from previously excavated sites in Northwest Washington (USA) from the Makah and Quilleute Tribal Reservations, including the Ozette site [28], a shell midden deposit on the Makah Tribal Reservation, and a shell midden on the Quilleute Tribal Reservation (Table 1, Figure 1). Excavations took place between 1971 and 2005. All bones were dated based on previously-established site provenience [28] or AMS-^14^C dating at Lawrence Livermore National Laboratory (Livermore, CA) after correction for the marine reservoir (North Pacific surface reservoir) [29], [30], [31].

**Table 1:** Ancient samples: sampling locations, units and dates in calendar years based on direct radiocarbon dating of bones (samples in italics) or of associated shell middens.

Sample	Site	Date (ybp)
**BAL4**	45CA24B70	300–500
**BAL5**	45CA24B70	150–250
***BAL6***	45CA24B70	370–490
**BAL12**	45CA24B70	300–400
**BAL15**	45CA24B70	300–500
**BAL16**	45CA24B70	300–500
**BAL17**	45CA24B70	150–250
***BAL18***	45CA24B70	280–370
**BAL19**	45CA24B70	150–250
**BAL20**	45CA24B70	150–250
***BAL21***	45CA24B70	260–380
***BAL23***	45CA24B70	310–420
***BAL24***	45CA24B70	430–520
***BAL25***	45CA24B70	320–420
***BAL28***	45CA400	2450–2690
**BAL37**	45CA23	660–880

45CA24B70  =  Ozette site; 45CA400  =  Shell midden deposit; 45CA23  =  Shell midden on Quilleute Indian reservation.

### DNA Extraction, Amplification and Sequencing

DNA extraction and amplification were performed under strict ancient DNA contamination control measures (see “Authentication” below). The surface of each sample was removed via sanding and ca. 0.1–0.3 g of bone was removed using a dremel tool. Each subsample was ground into a fine powder and incubated overnight at 55°C with 1.25 mL of extraction buffer (0.5 M EDTA at pH 8.0, 0.5% SDS and 0.5 mg/mL proteinase K) in a 1.5 mL tube. DNA was extracted using Qiaquick DNA Extraction columns (QIAGEN) according to manufacturer’s instructions.

We amplified four overlapping fragments of the mitochondrial control region sized 180–250 bp (Table 2). First, a 182 bp fragment was amplifed using primers F22 and R258 as described in [32] and sequenced to determine species identity. We amplified all samples identified as gray whales at three additional fragments. Amplification conditions were as follows: 0.1 µM each primer, 2.5 mM MgCl_2_, 0.2 mM dNTP, 1.5 mg/mL spermidine, 2.5 µL DNA template, and 1.25 U Amplitaq Gold (Applied Biosystems). Amplifications were performed on a BioRad cycler with the following profile: initial denaturation at 95°C for 12 minutes, 40 cycles of 94°C/30 s, 55°C/30 s, 72°C/40 s, and a final extension at 72°C for 10 minutes.

**Table 2:** Primers used in the ancient DNA analysis (5′-3′ direction).

Primer name	Sequence	Reference
dlpF22	CCACCATCAGCACCCAAAGC	[32]
dlpR258	TGCTCGTGGTGTARATAATTGAATG	[32]
ERdlpF1	CCCATAGTARTTAGTATTCCCCTGTG	This study
ERdlpR1	CACAGGGGAATACTAAYTACTATGGG	This study
ERdlpF2	CTTCACTACGGAAGTTAAAGCCCG	This study
ERdlpR2	CGGGCTTTAACTTCCGTAGTGAAG	This study
ERdlpF3	CAGCATGCCGCGTGAAACCAGCAACCC	This study
ERdlpR3	GGGTTGCTGGTTTCACGCGGCATGCTG	This study
ERdlpF4	GCAGGGATCCCTCTTCTCGCACCGG	This study
ERdlpR4	CCGGTGCGAGAAGAGGGATCCCTGC	This study

We purified all succesful amplification products using Qiaquick columns (QIAGEN). All purified products were sequenced in both directions on an ABI 3100 Genetic Analyzer. A subset of 20% of amplification products were cloned (with a minimum of 8 sequences per product) to determine whether exogenous amplicons were present, using ABI Topo kit. Sequences were cleaned, edited and aligned in Sequencher 4.0 (GeneCodes).

### Authentication

Ancient DNA extraction and pre-PCR procedures were performed under strict controls to minimize contamination risk and controls were included at each step to monitor contamination. Primers were designed specifically for cetaceans, and laboratories in which extractions and PCR were performed had never had any modern whale or cetartiodactyl DNA or tissues in the facilities. Ancient DNA extraction and pre-PCR procedures took place in a specialized facility, spatially isolated from facilities in which PCR, cloning and sequencing take place. The ancient DNA facility is equipped with positive airflow to prevent/minimize exogenous contaminants from entering the room, and overhead UV lamps to destroy non-target DNA. No researchers are permitted to enter the clean room within 24 hours of contact with facilities in which PCR occurs. Prior to extraction of DNA from ancient material, all surfaces were cleaned with Alconox detergent and a bleach solution (10–30%), and room, materials (including tubes, tips, pipettors, and foil) and reagents (excluding proteinase K) were UV-irradiated overnight. Extractions and PCR set-up were performed in a Class II laminar flow hood. Samples were stored in separate airtight plastic bags until use. Each sample represents a different individual because subsamples came from the same complete skeletal element, had different ^14^C dates, or were from different sites.

All extractions and amplifications included negative controls at a ratio of one control for every four samples. Multiple, overlapping amplifications with different primer pairs were used to confirm all SNPs. Amplifications were repeated for 20% of samples. As described above, 10% of amplified fragments were cloned and sequenced to determine the extent of contamination by exogenous DNA. In addition, 25% of gray whale samples were re-extracted and amplified independently by a separate laboratory. New gray whale haplotypes were deposited in NCBI with corresponding sample names (Accession numbers JQ910911–JQ910926).

### Genetic Diversity

Ancient sequences were aligned to previously published control region sequences for both eastern and western Pacific gray whales [17] using Sequencher 4.0 (GeneCodes). Haplotype diversity (*Hd*), the genetic diversity parameters Watterson’s *?* and nucleotide diversity (*π*), and Tajima’s D were estimated using DnaSP v.5 [33]. We measured genetic differentiation between sample sets using *F_ST_* [34], and derived 95% confidence intervals from 20,000 bootstrap replicates using the program Arlequin v. 3.1 [35].

### Coalescent Simulations and Demographic Analyses

To explore whether bottlenecks could result in observed patterns, we used a rejection-based approximate Bayesian computation (ABC) approach [36] with serial coalescent simulations. We simulated a range of demographic histories (including population bottlenecks of different sizes/timing and various pre-bottleneck sizes) and used an ABC framework to compare observed and simulated values of summary statistics to estimate the posterior probability distributions of demographic parameters. In these simulations, population size parameters were estimated in terms of female effective size (N_ef_), or the number of breeding females. To relate these estimates to previously published figures, we converted between effective female size and census size using three steps (see [4], [5] for additional details and rationale): 1) female effective size is converted to effective size (N_e_) by multiplying by 2 to account for males; 2) effective size (N_e_) is converted to all adults (N_T_) by multiplying by 2, and 3) Adult population (N_T_) is converted to census size (N), or the total number of individuals in the population including juveniles, by multiplying by 1.5.

**Figure 2:**
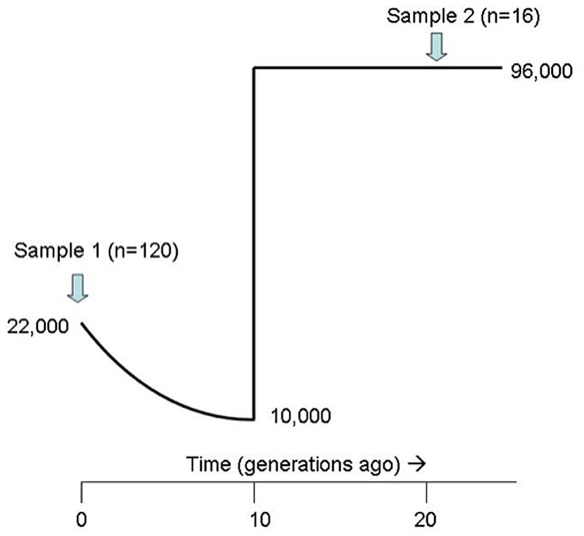
Simulated demographic scenario. The size of the ancient population is assumed to range from 20,000–116,000 (census size). The modern population is assumed to have a census size of 22,000. The size and timing of the bottleneck (pictured here at 10,000 individuals and 10 generations ago) were varied between 100–10,000 (census size) and 1–100 generations ago.

We varied demographic scenarios as follows. The time of the bottleneck was varied from 1–100 generations ago, prebottleneck size was varied from N_ef_ = 3333–19,333 (equivalent to N = 20,000–116,000) in the past, and minimum abundance at the bottleneck was varied from N_ef_ = 17–1667 (equivalent to N = 100–10,000 individuals) (Figure 2). The range of original abundance employed in the simulations was derived from today’s census size and an analysis of genetic diversity in nuclear introns of gray whales [5], and the range of bottleneck sizes was derived from the highest [18] and lowest [37] estimates available in the literature. Simulations use a generation time of 15.5 years, equal to the median age of reproductive females [38]. The molecular substitution model used (HKY+G) was selected using the program MODELTEST using Akaike Information Criterion (AIC) [39]. A range of mutation rates from 4.00–8.00×10^−8^ bp^−1^ yr^−1^ were employed based on the analysis of [27], which used cytochrome-b data to calibrate rate of substitution in the control region of gray whales. The method used to derive this rate, which is 2 to 4.4-fold faster than the phylogenetically derived rate (e.g. [40]), has been found to be consistent with results obtained in subsequent studies of mammalian rates [41], [42], [43]. To test the sensitivity of results to mutation rate, we also repeated the analysis using rates derived from Bayesian MCMC analysis of ancient and modern data (see below). We chose sample sizes and ages of samples to reflect our empirical dataset. Simulations were generated in Bayesian Serial SIMCOAL [44], [45] and rejection-based ABC was implemented in the statistical package R version 2.0 following the algorithm described in [13]. We performed 1,000,000 simulations with 1000 acceptances. We used five summary statistics (π_modern_, π_ancient_, *F_ST_* (ancient-modern comparison), Hd_modern_ and Hd_ancient_) to estimate posterior likelihoods for three parameters: 1) bottleneck time in generations (t_bot_); 2) minimum size of population (N_ef(bot)_); and 3) pre-bottleneck abundance (N_ef(prebot)_).

In addition, all ancient and 120 modern sequences were used to compare the likelihood of different demographic scenarios in a Bayesian MCMC analysis as implemented in BEAST v 1.5.3. MODELTEST [39] was used to determine the best-fitting substitution model. Based on these results, analyses were run using the HKY+G substitution model with a relaxed molecular clock (uncorrelated lognormal) in order to allow rates to vary among branches [46], and 30,000,000 iterations after a burn-in of 100,000 iterations, with sample ages used in the calibration and a uniform prior on mutation rate of 4.00–8.00×10^−8^ bp^−1^ yr^−1^. We selected the numbers of iterations and burn-in steps to ensure model convergence, and averaged results over five replicate runs. Both geneaologies and model parameters were sampled every 3000 iterations. Mixing and convergence were determined to be adequate based on the effective sample sizes (ESS) of each parameter, as evaluated in Tracer v. 1.3. We compared the following demographic models: constant population size, exponential growth, and Bayesian skyline plot (BSP) using 10 temporal groups. We compared support for models by calculating Bayes factors using the harmonic means of sampled marginal likelihoods for each model [47]. Additionally, BEAST v1.5.3 was used to assess levels of post-mortem DNA damage and take account of this damage in demographic analyses (see [48]). The potential for such damage to confound demographic analyses is an important consideration in assessing the ability of ancient or historical sequences to shed light on past population processes (e.g. [49]). However, Rambaut et al. [48] showed through simulations that when damage was measured and accomodated in aDNA analyses, evolutionary parameters and demographic reconstructions were correctly recovered.

Finally, we also evaluated past population dynamics using the Bayesian skyline plot (BSP) method of Drummond et al. [8]. In this method, a sample of gene sequences (including sequences sampled at different points in time) is used to estimate effective population size through time, using an MCMC sampling procedure. The method produces credibility intervals that incorporate both phylogenetic error and uncertainty inherent in reconstructing the coalescent process. However, using limited sequence data from a single locus can reduce the power of this method to detect population dynamics in the past [22]. To determine whether our ancient samples were adequate for detecting the signature of a bottleneck in Bayesian demographic analyses, we repeated the analyses on simulated datasets with identical ancient sampling but known demographic histories. We analyzed two demographic scenarios in which bottlenecks were assumed to have occurred at 800 or 1200 ybp (reducing the population from 96,000 individuals to 22,000). All other parameters (such as mutation rate and generation time) were identical to those used in the demographic simulations described above.

### Stable Isotope Analysis

In addition to assessing the stable isotope composition (δ^13^C and δ^15^N) of all ancient gray whale samples, we collected bone fragments from modern gray whale bones for the purpose of comparison. Fourteen gray whale bones were analyzed from the USNM collection, Smithsonian Institution. The majority of the USNM samples come from animals harvested in the 1960s and 70s at a California whaling station across different years [38], and are therefore likely represent a random subsample of the population. Bone fragments were demineralized in 0.5 N hydrochloric acid (HCl) for ∼12–15 hr at 5°C. The resulting material was treated repeatedly with a chloroform/methanol (2∶1) mixture to remove lipids and then lyophilized. Dried samples (∼0.5 mg) were sealed in tin boats and analyzed using a Carlo-Erba elemental analyzer interfaced with a Finnegan Delta Plus XL mass spectrometer (Geophysical Laboratory, Carnegie Institution of Washington). Results are expressed as δ values, δ^13^C or δ^15^N = 1000[(R_sample_/R_standard_)-1], where R_sample_ and R_standard_ are the ^13^C/^12^C or ^15^N/^14^N ratios of the sample and standard, respectively. The standards are Vienna-Pee Dee Belemnite limestone (V-PDB) for carbon and atmospheric N_2_ for nitrogen. Units are expressed as parts per thousand or per mil (‰). Within-run standard deviation of an acetalinide standard was ≤0.2‰ for both δ^13^C and δ^15^N values. As a control for the quality of collagen, we measured the [C]/[N] ratio of each sample; weight percent [C]/[N] ratios of all bone collagen samples were 2.8–3.2, within the theoretical [C]/[N] ratio of unaltered collagen [50]. We applied a correction to all carbon isotope values to account for the global decrease in the ^13^C proportion of atmospheric carbon dioxide (i.e., “Suess Effect”), due largely to fossil fuel burning, over the last 150 years. Based on ice core records [51], we applied a time-dependent δ^13^C correction to historic samples (1912–1975) of −0.005 per mil/year between 1860 and 1960 (n = 1), and −0.022 per mil/year since 1960 (n = 13). This resulted in relatively minor (mean  = −0.3‰) δ^13^C corrections for the modern samples since most of them (12/14, ∼85%) were collected prior to 1970.

## Results

We extracted and amplified DNA from 38 of 40 samples. Alignment of control region sequence with baleen whale sequences from NCBI showed that 16 of the 38 sequences grouped with gray whales (remaining sequences grouped with humpack, blue or sperm whales). No exogenous contaminants or mismatches were detected in any of the cloned sequences or sequences from independently extracted specimens. Blind resequencing of a subset (25%) of modern sequences did not yield any sequence discrepancies with NCBI data. Only genetic data from gray whales (383 bp) were used for the remaining analyses.

### Genetic Diversity

The level of haplotype diversity across ancient samples (Hd = 0.933) was comparable to that found in modern Eastern samples (Hd = 0.948) and higher than that of modern Western samples (Hd = 0.700) (Table 3). Nine haplotypes were obtained from the sixteen gray whale samples, including three haplotypes previously unobserved in either the eastern or western Pacific populations. These unique haplotypes differed by four (one individual), two (one individual) and one (two individuals) base pair changes from known haplotypes. All but one of these changes were transitions. Values of *<$>\scale 80%\raster="rg1"\<$>(S)* and nucleotide diversity (*π*) were also similar across modern and ancient eastern Pacific samples. Tajima’s D values were nonsignificant for all three sets of samples.

**Table 3:** Summary statistics (±SD) for ancient Eastern Pacific (EP) samples, Modern EP, and modern Western Pacific (WP) samples.

	N	N(H)	Hd	π	θ(S)	Tajima’s D
**Ancient EP**	16	9	0.933±0.035	0.0130±0.0016	0.0127±0.0053	−0.031
**Modern EP**	120	30	0.948±0.007	0.0191±0.0009	0.0189±0.0041	0.906
**Modern WP**	45	10	0.700±0.049	0.0187±0.0012	0.0190±0.0045	1.392

N = number of samples; N(H) = number of haplotypes; *Hd* = haplotype diversity, *θ(S)* = Watterson’s theta [76]; *π*  = nucleotide diversity [77]. Values of Tajima’s D were nonsignificant for all samples (p>0.10).

**Figure 3:**
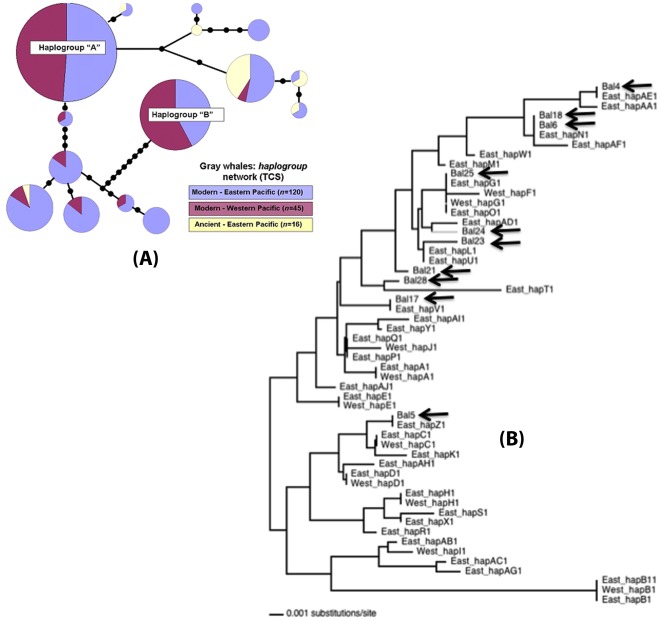
Phylogenetic network and tree constructed from modern and ancient gray whale haplotypes. (**a**) Haplogroup network for ancient eastern Pacific and modern eastern and western Pacific samples (constructed in TCS [52]). Haplogroups were defined by grouping together sequences with one or zero differences. (**b**) Neighbor-joining (midpoint-rooted) tree using ancient and modern haplotypes from PAUP* [53]. The HKY85 model [75] was used to correct genetic distances. Ancient samples have the prefix BAL and are denoted with an arrow. Each haplotype is represented only once in the tree.

Both a haplogroup network constructed using TCS [52], and a neighbor-joining tree constructed using PAUP* [53] show that ancient samples are not distributed randomly across the distribution of modern eastern Pacific samples, but cluster in one part of the network or tree (Figure 3a, 3b). Significant differences in haplotype frequencies were observed between each pair of samples (p<0.001). The observed *F_ST_* value between modern eastern and ancient eastern was 0.1004 (95% CIs: 0.0640–0.1344). The difference between modern western samples and ancient sequences (*F_ST_* = 0.2794) was greater than the difference between modern eastern and western sequences (*F_ST_* = 0.1125).

### Coalescent Simulations and Demographic Analyses

**Figure 4:**
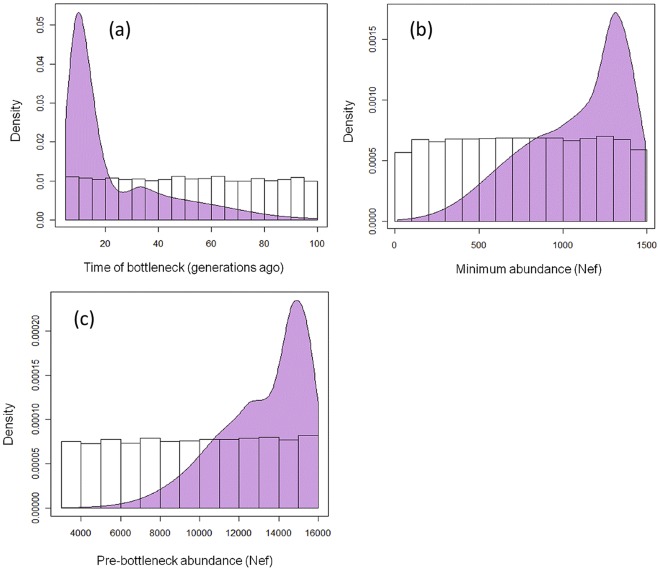
Posterior density distributions for Approximate Bayesian Computation results. Based on ancient eastern Pacific and modern eastern samples (shaded area) and prior uniform sampling distributions based on one million iterations for (a) time of bottleneck in generations (t_bot_); (b) minimum female effective population size at bottleneck (N_ef(bot)_); and (c) pre-bottleneck female effective population size (N_ef(prebot)_).

Posterior density curves and prior distributions for the three parameters of interest are shown in Figure 4. The maximum a posteriori estimate was for a bottleneck time 6 generations ago (90% highest posterior density interval (HPD) = 5–60 generations). Estimates for minimum abundance (N_(ef)bot_) and pre-bottleneck abundance (N_(ef)prebot_) were translated from units of female effective size (N_(ef)_) to census size (N) using conservative factors to account for sex ratio, the ratio of breeding adults to all adults and the ratio of juveniles to adults [5]. This resulted in maximum a posteriori estimates of N_bot_ = 9,070 (90% HPD = 3,750–9,740) and N_prebot_ = 100,670 (90% HPD:59,940–111,550).

Bayesian MCMC methods as implemented in BEAST can also be used to measure mutation rates directly when ancient data are available [54]. These methods can produce upwardly biased estimates of mutation rates in populations that deviate from simple demographic histories, especially in cases where population bottlenecks have occurred or population structure is or was pronounced [55], as is likely to be the case for the population considered here. However, in order to test the sensitivity of the ABC analysis to a range of rates, we implemented this method to derive control region rates. The Bayesian MCMC method gives a rate of 0.032–0.194 (95% HPD; mean 0.11) substitutions/site/My when applied to this dataset, a wide range that overlaps with the full range of rates used in this study. This broader range of mutation rates with a higher mean value produces a wider range of Ne values with smaller MLEs, resulting in MLEs of N_bot_ = 8,890 (90% HPD = 2,500–9,610) and N_prebot_ = 69,890 (90% HPD:41,220–109,210), and T_bot_ = 13 (90% HPD = 9–68).

**Figure 5:**
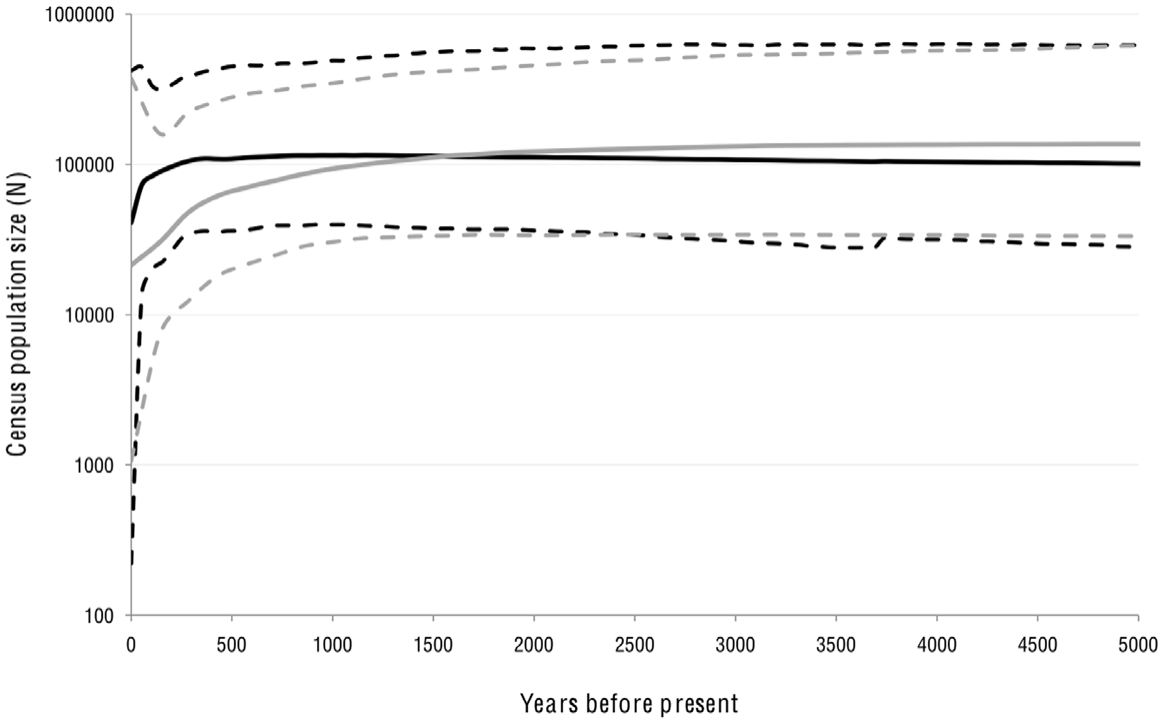
Bayesian skyline plots using empirical ancient and eastern Pacific modern datasets. (black solid line = median; black dashed lines = 95% highest posterior density intervals (HPDIs)), and for a simulated dataset in which bottleneck from 96,000 to 22,000 individuals occurred 1200 ybp (gray solid line = median; gray dashed lines = 95% HPDIs). BSP results were averaged across five replicate runs. NB: The BSP analysis used here assumes a single panmictic population.

We used ancient and modern sequences to compare the likelihood of different demographic scenarios in a Bayesian MCMC analysis. A Bayes factor analysis of sampled marginal likelihoods for each model indicated some support for the Bayesian skyline plot (BSP) model over the demographic models of constant or exponential growth (BF>2). The skyline population trend is also consistent with a recent decline (Figure 5). BSP analyses using simulated datasets showed broad declines that were consistent with the bottleneck dates simulated (e.g. roughly 1200 ybp). However, for both the real dataset and simulated datasets, confidence intervals are extremely wide and the skyline plots do not successfully recreate the most recent fine-scale population dynamics over the past ∼150 years (population bottleneck followed by regrowth). The mean rate of post-mortem damage estimated in BEAST was 2.37×10^−8^ errors per base pair (95% highest posterior density interval: 6.99×10^−12^, 7.25×10^−8^). This estimated rate is lower than several other D-loop datasets derived from samples of comparable (though generally older on average) age, such as ox (4–8 kya, HPD: 3.87×10^−7^–8.57×10^−4^), moa (1–6 kya, HPD: 1.75×10^−5^ to 3.58^−3^), and musk ox (0–44 kya, HPD: 9.81×10^−8^–1.91×10^−3^) [56].

### Stable Isotope Analyses

**Figure 6:**
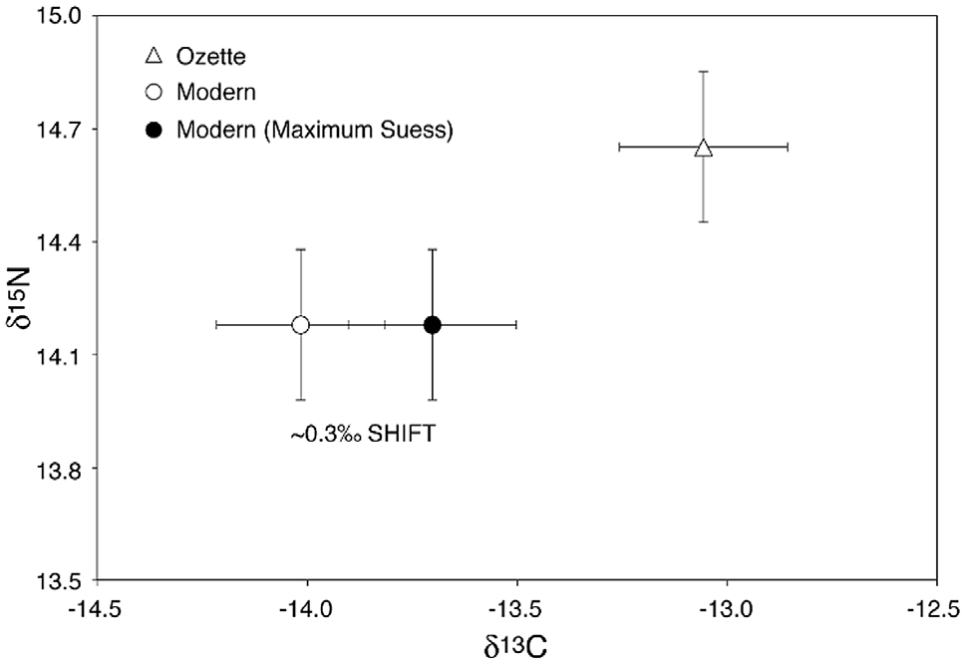
Mean stable isotope values for modern (USNM), modern corrected (USNM (maximum Suess)) and ancient (Ozette) samples; error bars represent standard errors. No significant overall difference between ancient and modern samples is observed once modern samples are corrected for the Suess effect. The Suess effect results in an average shift in δ^13^C of 0.3%.

Ancient gray whales had significantly higher mean δ^13^C values (ANOVA or pooled T-test, p<0.05) than the modern whales (Figure 6); there were no differences in mean δ^15^N values. The mean (±SE) δ^13^C value for Suess corrected modern (n = 14) and ancient (n = 16) gray whales was −13.7 (±0.2) and −13.1 (±0.1) respectively. The mean (±SE) δ^15^N values for modern and ancient samples were 14.2 (±0.2) and 14.7 (±0.2), respectively. For modern samples, for which age and sex were sometimes known, no obvious effects were observed based on these factors (though small sample size prevents a thorough analysis).

## Discussion

Ancient gray whale sequences show high genetic diversity, but this diversity is not randomly distributed with respect to today’s haplotype distribution (Figure 3). There are at least two potential causes for this non-random distribution: past population structure, and a large demographic bottleneck that resulted in the reshuffling of haplotype frequencies.

Population structure in the past could result in significant genetic differences between modern and ancient whales. All ancient samples were collected from the same geographic area (the Olympic Peninsula) and were likely caught in or outside of the Strait of Juan de Fuca, raising the possibility that this area might have harbored a genetically unique population in the past. This possibility is particularly worth exploring because a small subset of the modern gray whale population uses the Strait of Juan de Fuca and Puget Sound as a summer feeding ground, whereas the large majority of gray whales travel north to the Bering Sea and northward to feed [25]. Photoidentification data shows that at least some of these individuals return year after year to the area to feed (Calambokidis et al. 2002). Though an earlier genetic study found no evidence that these animals represented a unique population [23], a more recent analysis detected slight but significant differentiation between the southern feeding aggregation and the gray whale population as a whole [57].

To explore pre-whaling feeding ecology and test the hypothesis of population structure in the past, we investigated the stable isotope signature of the ancient whales and a set of modern gray whale bones from the USNM collection. We presume the USNM whales, which were mostly collected at a whaling station near Richmond, California in the 1970s, represent a random subset of the population because whales were taken in different years along their central migration route, and thus would carry the isotopic signature of the primary northern feeding grounds. Gray whales are unique among large cetaceans in that they migrate and feed close to shore, typically <80 km [38], and therefore we would not expect distance from shore to be a confounding factor in interpreting isotopic results. If in fact the ancient whales represented a genetically distinct resident aggregation in the past, the most likely scenario is that these whales were feeding in the Strait of Juan de Fuca and Puget Sound, which would result in different isotopic signature due to differences in foraging latitude. Phytoplankton and dissolved organic matter δ^13^C and δ^15^N values are negatively correlated with latitude in the northeast Pacific Ocean [58], [59], [60]; temperate latitude systems (e.g., California Current) have higher isotope values by ∼1–2% than high latitude systems (e.g., Bering Sea). As such these isotopes have been used extensively to examine differences in foraging latitude in modern and ancient marine mammals [14], [15], [61], [62], After correcting for the Suess effect, we found slight but significant differences in mean δ^13^C values between the two groups; mean δ^15^N values were not significant. Assuming ancient and modern groups forage at similar trophic levels, the overall isotopic pattern is in agreement with that expected if ancient Ozette gray whales foraged in lower latitude waters than the modern group, which is known to forage at high latitudes in the Bering Sea. Thus, it remains possible that at least a subset of these whales were occasional summer residents in the area, particularly in light of the recent analysis by [57]. However, the small observed isotopic differences in δ^13^C and δ^15^N and small sample sizes suggest that drawing a firm conclusion about geographic structure from these isotopic data would be premature. Finally, it is also possible that the ancient whales from Ozette represent a genetically unique population, due to structuring along another ecological axis other than feeding. Further tests of the hypothesis of population structure in the past will require additional ancient samples from this region and new locales.

We tested the second possible cause for nonrandom distribution of ancient haplotypes, a demographic bottleneck, using serial coalescent simulations and rejection-based Approximate Bayesian Computation approach. We selected demographic scenarios used in coalescent simulations by using a range of values of population size in the past and today from census [63] and genetic data [5], and exploring potential bottleneck dynamics that might have occurred in the interim. These analyses demonstrate that a subset of demographic scenarios are most likely to produce the observed summary statistics in modern and ancient samples. In particular, the observed *F_ST_* value can result from a bottleneck followed by rapid population growth. Simulations demonstrate that, as expected, more severe bottlenecks create higher *F_ST_* values. Results indicate highest support for a population bottleneck that between 5–60 generations ago (90% HPD), with a maximum likelihood estimate of 93 years or 6 generations, which roughly corresponds to the end of the central period of commercial whaling (Figure 4). Little is known about the size of the gray whale population during the height of industrial whaling around 1890, though it is known that the population was determined to be “commercially extinct” [16]. Previous estimates vary from 150 based on visual census [37] to 10,000 based on population models [18]. Simulation results give an MLE of 9,070 (90% HPD: 3,750–9,740), much closer to the latter value. This larger estimate is in agreement with the rapid growth of the gray whale population during the last half of the 20^th^ century, and brings estimates of pre-whaling abundance from whaling records (which reflect whales killed in addition to the number of individuals remaining at the bottleneck) into slightly closer alignment with those from genetics. The posterior distribution of pre-bottleneck census size (MLE = 100,670, 90% HPD:59,940–111,550) is higher than those estimated from whaling records, and corresponds to the distribution of 96,000 (78,000–116,000) previously estimated from a separate genetic dataset (nine nuclear introns and cytochrome*-b*; [5]).

In addition to the simulation approach, we used a Bayes factor analysis to determine which demographic model (constant, exponential growth, or Bayesian skyline plot) provided the best fit to the data. The BSP provided a better fit than the other two models (BF>2), suggesting a population decline. The skyline plot analyses based on modern and ancient control region sequences are consistent with a recent decline, and there is no indication of an earlier major decline. Though the possibility remains that our dataset violates the assumption of panmixia, previous studies indicate that skyline plots are relatively robust to such violations [8], [11]. The BSP analysis also successfully reconstructed earlier hypothetical declines in simulated datasets using the same sample size and age distribution as in our empirical dataset, indicating that if a decline from 100,000 to 20,000 individuals occurred earlier in the Holocene, we would expect to detect it with our dataset. However, in both cases credibility intervals are large due to small ancient sample size and uncertainty inherent in the coalescent process and phylogenetic reconstruction, limiting the inferences we can draw from these results. In addition, previous analyses of ancient DNA datasets using Bayesian skyline plots (e.g. [8], [11]) and bowhead whales [64] indicate that this methodology was unable to reconstruct very recent declines or bottlenecks. Additional loci and ancient samples would be needed to gain enough statistical power to quantify very recent bottlenecks with confidence.

Estimating demographic parameters from genetic data requires the estimation of evolutionary rates and other uncertain factors. Recent studies have suggested mtDNA mutation rates estimated from phylogenetic data are inappropriate for intraspecific studies because of time dependency of molecular rates (older calibration points produce slower clock rates) [54], [65]. In this study, we addressed this problem by using a range of evolutionary rates derived from intraspecific calibration of the control region based on variation at a linked locus [27]. This method utilizes more recent calibration points and is thus better able to detect multiple hits/homoplasy, a common feature of the mammalian control region that may contribute to differences between pedigree-based and phylogenetic rate estimates [66]. Bayesian MCMC methods have been used to measure mutation rates directly when ancient data are available [54], but simulation studies found these methods can overestimate the true rate for populations in which bottlenecks have occurred or those with pronounced structure [55] (however, it is important to note that some of the simulation scenarios used in the latter study included non-representative sampling). In addition, a recent study found that some ancient DNA datasets, including bowhead whale, produced artifactual rate estimates as a result of low information content among other factors including sequence ages [67]. For this reason, we consider the range of rates derived from intraspecific calibration [27] to be the best available estimates for use in this analysis, in the absence of a molecular rate curve [46] for baleen whale species.

Additional uncertainties in the estimates of total population size (N) arise from other parameters needed for the analysis, including the ratio of breeding adults to total adults (Ne/N), generation time, the sex ratio and the ratio of juveniles to adults. While gray whale-specific estimates exist for the latter two values, Ne/N is very poorly known for most species [68]. A review of empirical studies suggested that the number of breeding individuals in a population is typically an order of magnitude below the total number (averaging 0.10–0.11), and that Ne/N rarely falls above 0.5 in natural populations [69]. Theoretical analyses suggest that Ne/N approaches 0.5 in most populations with constant size [70]. Factors that can reduce Ne/N include uneven sex ratios, population bottlenecks and variance in reproductive success (e.g. [71], [72]). In this analysis, we used a conservative estimate of Ne/N (0.5), which will produce smaller estimates of total population size; however, it is important to recognize the additional uncertainty introduced by this calculation. While empirical and theoretical studies indicate that this value is unlikely to be an underestimate for gray whales, it is possible that the true Ne/N ratio might be much smaller. Likewise, generation time is difficult to measure with precision in wild populations, and may not necessarily be stable across evolutionary time scales. In this analysis, we use a standard definition of generation time, calculated as the mean age of reproductive females, assuming no decline in fecundity with age [68]. A decline in fecundity with age would reduce the estimated generation time, causing a proportional increase in the population size estimated from genetic data. If, on the other hand, the average generation time of gray whales across the last several thousand years was greater than estimated here (for example if whaling caused average generation time to decrease), it would cause a proportional reduction in DNA-based *N_e_* estimates. These caveats regarding life history parameters underscore the uncertainties associated with inferring population size and dynamics from genetic data, which have been discussed in depth in previous works (e.g. [6], [7], [73]).

Overall, the genetic evidence presented here supports the hypothesis that gray whales experienced a major population decline, and that this reduction occurred recently. Stable isotope results show only very slight differences between ancient and modern whales, indicating the hypothesis of population substructure in the past around the area of the Olympic peninsula/Vancouver Island remains a possibility and warrants further investigation using larger sample sizes. Though our ability to infer what was surely a complex demographic history is limited by the number of ancient samples available and large uncertainties associated with the coalescent and evolutionary processes, these first ancient data for gray whales demonstrate the value of paired genetic and isotopic studies of ancient samples, showing that a population bottleneck can result in significant genetic differentiation between ancient and modern samples without requiring spatial structure. Both demographic simulations and coalescent analyses indicate that genetic data are consistent with a recent bottleneck and a pre-bottleneck size of >ca. 60,000. Recent models of gray whale carrying capacity during the Pleistocene suggest that enough benthic habitat existed to support a population of this size [74]. Future exploration of the impacts of population structure (particularly between eastern and western populations) and analysis of whaling records may be informative regarding the unresolved discrepancy between whaling estimates and genetic estimates of historic abundance. Understanding the causes and extent of the decline in marine species is important to their future management and aids in reconstructing the past states of ocean ecosystems. The analyses presented here corroborate an emerging body of evidence demonstrating historic baselines for many marine populations much larger than previously estimated.
